# Effects of error on dose of target region and organs at risk in treating nasopharynx cancer with intensity modulated radiation therapy

**DOI:** 10.12669/pjms.321.9218

**Published:** 2016

**Authors:** Guangsheng Liu, Sumei Zhang, Yuzhuo Ma, Qingyuan Wang, Xingxiu Chen, Lingling Zhang, Fengmei Ma

**Affiliations:** 1 Guangsheng Liu, Binzhou People’s Hospital, Shandong, China; 2 Sumei Zhang, Binzhou People’s Hospital, Shandong, China; 3 Yuzhuo Ma, Binzhou People’s Hospital, Shandong, China; 4 Qingyuan Wang, Binzhou People’s Hospital, Shandong, China; 5 Xingxiu Chen, Binzhou People’s Hospital, Shandong, China; 6 Lingling Zhang, Binzhou People’s Hospital, Shandong, China; 7 Fengmei Ma, Binzhou People’s Hospital, Shandong, China

**Keywords:** Nasopharynx cancer, Intensity modulated radiation therapy, Setup error, Physical dosimetry

## Abstract

**Objective:**

To measure setup error of head and neck neoplasm in radiotherapy and discuss over effects of error on physical dose acting on target region and organs at risk of nasopharynx cancer (NPC) patients treated with intensity modulated radiation therapy (IMRT).

**Methods:**

A total of 152 patients who developed head and neck neoplasm and received IMRT were randomly selected. Through comparing digital portal image and digital reconstruction image, we measured setup error, calculated expanding margin from clinical target volume (CTV) to planning target volume (PTV) and analyzed whether there was rules between setup error and treatment time. Additionally, 20 cases of NPC were selected. Three-dimensional error was simulated in planning system. Dose distribution was recalculated and a series of dose parameters of target volume and OAR were analyzed.

**Results:**

Setup error in left-right, head-feet and ventral-dorsal direction was (-0.62±1.46) mm, (-0.41±1.54) mm and (-0.31±1.67) mm respectively. Regarding limit value, the maximum and minimum value in left-right direction, head-feet direction and ventral-dorsal direction was 2.70 mm and -6.00 mm; 3.00 mm and -5.00 mm, 5.00 mm and -7.50 mm. Expanding margin from CTV to PTV was 2.26 mm, 1.88 mm and 1.97 mm in left-right direction, head-feet direction and ventral-dorsal direction.

**Conclusion:**

During IMRT, only when setup error is controlled below 3 mm can sharply reduce the damage caused by radiation to normal tissue; therefore, quality security and control of electronic portal imaging device need (EPID) to be improved.

## INTRODUCTION

Nasopharynx cancer (NPC), the most common head and neck malignant tumor, is usually treated by radiotherapy as anatomic sites are close to multiple vital organs.^1^ On this account, cure rate and survival rate are directly determined by radiation technology. In recent years, intensity modulated radiation therapy (IMRT) as the mainstream radiation technology is gradually used in clinic. Differing from conventional radiotherapy, IMRT is able to provide radiation on different target tissue regions as required by altering radiation technology.^2^ When IMRT is applied in treating NPC, radiation dose can concentrate on target region extremely, which can ensure less radiation on organs beside target region, improve efficiency in killing tumor cells and enhance local control rate of tumor and survival rate.^3^

In precise radiation treatment, movement or deformation of organs as well as setup error in each treatment can result in significant difference between actual dose and planned dose of target region and surrounding important normal tissues. Therefore, setup error plays a key role in IMRT.^4^ IMRT as a kind of the most advanced technologies can improve local control rate of tumor and survival rate. If influence caused by setup error fails to be eliminated, the advancement of IMRT cannot be fully displayed. Thus it is quite necessary to accurately measure setup error and analyze effect of setup error on physical dose.^5^ Wang F and Yang SS found imaging of head and neck was more accurate than breast and pelvic cavity by electronic portal image device (EPID) and comparing that with standard imaging in horizontal and vertical direction.^6^ Xia TY reported that, average error of fixed setup of head and neck neoplasm was (2.47±0.96) mm.^7^

This study explored setup error of head and neck neoplasm in IMRT, simulated position difference with error value in planning system and then analyzed setup error on target region and OAR.

## METHODS

One hundred fifty two patients with head and neck neoplasm treatment were selected in our hospital between July 2012 and September 2014. They aged from 4 to 68 years. Among them, 114 cases were NPC and the other 38 cases were other head and neck neoplasm such as laryngocarcinoma, brain tumor, parotid tumor, etc. Twenty patients who were pathologically confirmed having low-differentiated NPC were randomly selected, including 16 meals and 4 females. They aged 37 to 67 years (average 54.5 years). Of 20 cases, 6 cases were stage II, 10 cases were stage III and 4 cases were stage IV. Number of cases with T1~T4 was 2, 8, 8 and 2 respectively; N0~N3 was 2, 10, 6 and 2. The study has been approved by the medical ethics committee and written consent has been obtained from all patients.

### Image acquisition and error comparison

DRR was regarded as reference used for error analysis. Top of the head to 3~4 cm below clavicle was scanned by CT plain scan. Thickness of layer was set as 5 mm and space between layers was set as 5 mm. The obtained CT images were transmitted to planning system through network system. Reversed IMRT scheme and position verification plan were designed. Verification field included anterior field and left-side field. DDR obtained were stored in the image workstation.

Images shot by EPID were regarded as reference used for error comparison. Parameters of the accelerator were set based on verification field plan called by radiotherapy network system. Afterwards, EPID was started to shoot 0° and 90° portal image and the images obtained were stored in the image workstation. Then DRR and EPID images were analyzed by image processing software Vision 6.1 to compare position of the same anatomical structure in two images.

Coordinate system mentioned in 62th report released by International Commission on Radiation Units and Measurements was used in this study to analyze error. Vector was used to expressing offset in different direction. X stands for left-to-right direction and left direction is positive; Y stands for head-to-toe direction and head direction is positive; Z stands for venter-to-dorsum direction and venter direction is positive. Setup error of all patients in 3D direction was recorded. Systematic error Σ was expressed by average of all errors and random error σ was expressed by standard deviation of all errors.

### Simulation of setup error

Limit value of setup errors in all directions were taken as experimental value. Tumor of 20 patients was confirmed with CT in combination with magnetic resonance imaging (MRI). Primary focus in nasopharynx was called as gross tumor volume GTV1; positive lymphonodus in neck was called as GTV2; soft tissue or lymphonodus around tumor was called as clinical target volume CTV1; lymphonodus prophylactic irradiation area was CTV2.

Taking final treatment scheme of every patient as template, we copied radiation field into planning CT image without any change. Then value of planning isocenter coordinate was altered in some direction to simulate inaccuracy of position of patients in actual treatment. Six new plans were set up, and their centers moved towards six different directions. The other conditions such as field shape, gantry angle and prescribed dose remained the same. Dose distribution were recalculated. Monitoring unit which acquired every field of position movement plan was kept consistent with treatment plan.

### Observation index

A series of dose parameters of target region and organs at risk in template plan and position movement plan were made into a dose volume histogram (DVH). Statistical comparison was performed to analyze effects of setup error on physical dose acting on target region and organs at risk.

### Statistical analysis

SPSS19.0 statistics software was used to process data. Measurement data were expressed as Mean±SD. Data was compared using t test and one-way analysis of variance. Difference was considered to be statistically significant if P<0.05.

## RESULTS

**Fig.1:**
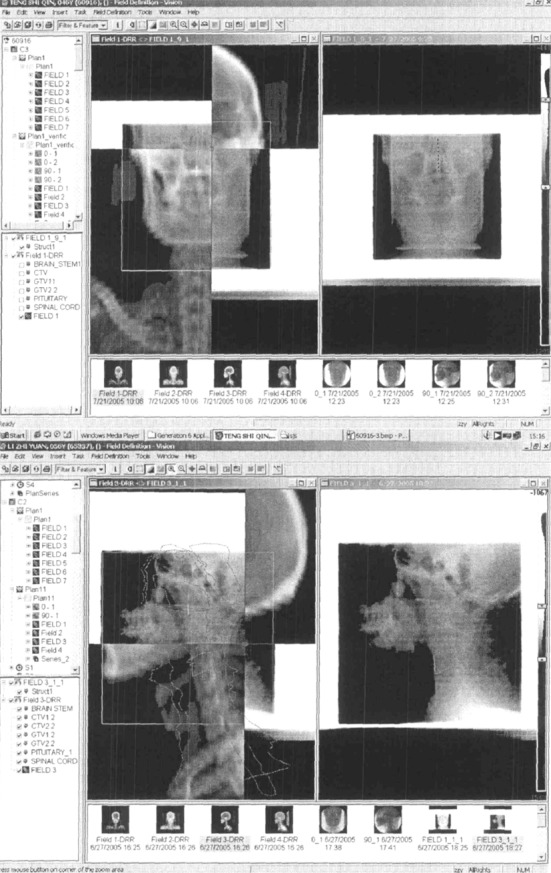
Image comparison result.

The basic interface of image analysis is shown in Fig.1. In the interface, the left image is comparison of frontal field images and the right one is comparison of profile field images. In actual measurement, error in left-to-right direction is obtained by comparing nasal septum and eye socket in images; error in head-to-toe and venter-to-dorsum direction is obtained by comparing centrum and vertebra posterior border. We only analyzed linear error in three dimensional directions, without considering rotation error or deformation error. That is because head and neck neoplasm region is bony structure with little distortion.

### Statistics of setup error

As shown in Table-I, the first group of data refer to errors between images acquired by EPID and DRR of 152 patients in the first time of treatment and the second group of data are errors between images acquired by EPID and DRR (not include measurement data obtained in the first time of treatment); the third group of data are errors between images acquired by EPD and DRR (include all measurement data). Positive and negative sign stands for vector property.

**Table-I:** Statistics of setup error of all patients (mm)

Direction	System error Σ(mm)	Random error σ (mm)	Setup error		Setup error ≥ 3mm(%)

			Maximum	Minimum	
X1	-0.48	1.58	2.70	-6.00	6.5
Y1	-0.42	1.42	2.50	-5.00	5.4
Z1	-0.071	1.60	5.00	-5.00	9.3
X2	-0.75	1.28	2.00	-3.50	5.7
Y2	-0.42	1.68	3.00	-5.00	14.2
Z2	-0.55	1.71	5.00	-7.50	8.6
X3	-0.61	1.47	2.70	-6.00	6.9
Y3	-0.42	1.55	3.00	-5.00	9.4
Z3	-0.32	1.66	5.00	-7.50	8.9

### Calculation of expanding margin from CTV to PTV

Expanding margin from CTV to PTV was calculated using formula (2Σ + 0.7σ) mm. The third group data in Table-I was used for calculation and finally we got 2.26 mm for X, 1.88 mm for Y and 1.97 mm for Z.

### Analysis of effect of dosage

### Simulation of setup error

We took one patient as an example. In six copy plans, the patient was moved according to extreme value of setup error, i.e., move 3 mm toward left side, 6 mm toward right side, 3 mm toward head, 5 mm toward toe, 5 mm toward venter and 8 mm toward dorsum. In planning system, setup error could also be simulated by altering isocenter coordinate of original plan, i.e., move 3 mm toward right side, 6 mm toward left side, 3 mm toward toe, 5 mm toward head, 5 mm toward venter and 8 mm toward dorsum. Then dose distribution was recalculated.

### Display of dose lines

**Fig.2:**
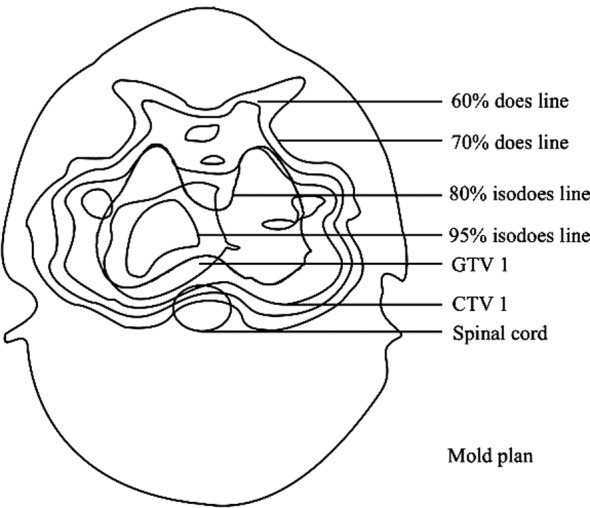
Mold plan.

Fig.2 shows isodose lines of center layer in treatment plan of one patient. 60% dose line borders spine cord and 70% dose line closely adheres to spine cord. It shows that, dose around spine cord is within the allowed range; the middlemost target region GTV1 is fully surrounded by 95% dose line; CTV1 is surrounded by 80% dose line, but certain margin exists. It suggests that, target regions were fully radiated.

### Analysis of physical dose

In Table II and III, the difference of the influence of setup error on the lowest dose of GTV1 and GTV2 was significantly different; but difference of average dose had no statistical significance is highlighted. For minimum dose of CTV1 and CTV2, difference of some items were statistically different. As to OAR, only several parameters of spinal cord had statistically significant difference; brainstem, crystal and parotid gland all had only one parameter with statistical significance.

**Table-II:** Setup error.

Direction	Systematic error Σ (mm)	Random error σ (mm)	Extreme value of setup error (mm)	
			Maximum	Minimum
X	-0.62	1.46	2.70	-6.00
Y	-0.41	1.54	3.00	-5.00
Z	-0.31	1.67	5.00	-7.50

**Table-III:** Statistical analysis for dose parameters of target region.

Target region	Parameter	Template	Move left	Move right	Move toward head	Move toward toe	Move toward venter	Move toward dorsum
GTV1	Minimum dose	<0.0001	*	#	#	#	#	#
	Average dose	0.1088	*	*	*	*	*	*
GTV2	Minimum dose	<0.0001	*	#	#	#	#	#
	Average dose	0.0022	*	*	*	#	*	*
CTV1	Minimum dose	<0.0001	*	#	*	*	*	#
CTV2	Minimum dose	<0.0001	*	#	*	#	*	#

* stands for P>0.05;

# stands for P<0.05.

## DISCUSSION

NPC, the most common malignant tumor in China, can severely threaten patients’ lives once it occurs. 8 The preferred therapy for NPC currently is radiotherapy because surgical treatment is difficult to be carried out on the special lesion locations. NPC is highly sensitive to radiotherapy and moreover requires high dose, radiation area and radiation method.^9^,^10^ To control NPC better and increase survival rate, IMRT is gradually introduced into treatment of NPC.

IMRT, a three-dimensional treatment technology, is based on high-resolution CT, MRI or positron emission tomography-computed tomography (PET-CT).^11^,^12^ It can accurately position scope of risk area and distribute concentrated dose, thereby preventing normal tissue from radiation. For head and neck neoplasm, setup error has large influence on margin from CTV to planing tumor volume (PTV); therefore, accurate radiotherapy for head and neck neoplasm is usually affected by uncertainty of target region.^13^,^14^ Scholars and experts have gradually shifted their attention on how to reduce setup error in radiotherapy and confirm target margin in 3D direction.

Setup error refers to deviation between reference position and actual treatment position.^15^ Actual treatment position can be accurately confirmed through portal image, whereas reference position can be confirmed through DRR. Setup error can be acquired by comparing difference of position of the same structure on two images.^16^,^17^ As shown in Table-I, systematic error was below 1 mm, indicating the high precision of the equipment; and random error approximated to 1, indicating the high repeatability of every setup. That suggests that, random error is a key factor inducing setup error.

**Table-IV:** Statistical Analysis for dose parameters of organs at risk

Normal tissue	Parameter	Template	Move left	Move right	Move toward head	Move toward toe	Move toward venter	Move toward dorsum
Spinal cord	D1	<0.0001	*	#	*	*	#	#
Brainstem	D10	<0.0001	*	*	*	*	#	*
Optic nerve	D1	0.6316	*	*	*	*	*	*
Crystal	D1	<0.0001	*	*	*	#	*	*
Optic chiasma	D1	0.9476	*	*	*	*	*	*
Left parotid gland	D50	<0.0001	*	#	*	*	*	*
Right parotid gland	D50	<0.0001	*	#	*	*	*	*

* stands for P>0.05;

# stands for P<0.05.

Effect of setup error higher than 3 mm on minimum dose of target regions had statistically significant difference. For spinal cord and brainstem, effect of error higher than 3 mm in ventral-dorsal direction on dose also had significant difference. In IMRT for treating prostatic cancer, Ahmad S et al. once applied extreme value of error in head-feet direction 5 mm into planning system, recalculated dose distribution and observed changes of dose of target region and OAR; finally, they found that, average dose of target region and radiation dose of OAR both increased.^18^ We applied extreme value of error into planning system. Besides observing whether the error resulted in increase of dose, we further statistically analyzed whether changes of dose caused by error had statistical significance. Parameters with statistically difference indicates errors in those directions should be reduced, whereas parameters without statistically difference suggested there was no severe dose deviation caused by setup error during IMRT.

## CONCLUSION

In conclusion, high survival rate of NPC patients and local control of NPC is possibnle in IMRT if setup error is kept below 3 mm. This work improves people’s understanding on quality guarantee and control of IMRT in treating head and neck neoplasm and also leads to reliable clinical guidance for accurate radiotherapy on tumor. But this study has some limitations. For example, though software was used to compare error in image workstation, the outline of anatomical tissue was drawn by people which can lead to difference.

## References

[1] de Boer HC, van Sörnsen de Koste JR, Creutzberg CL, Visser AG, Levendag PC, Heijmen BJ (2001). Electronic portal image assisted reduction of systematic set- up errors in head and neck irradiation. Radia Oncol Biol Phys.

[2] Kam MK, Chau RM, Suen J, Choi PH, Teo PM (2003). Intensity-modulated radiotherapy in nasopharyngeal carcinoma: dosimetric advantage over conventional plans and feasibility of dose escalation. Int J Radiat Oncol Biol Phys.

[3] Wang XC, Han F, Yang F, Liu XQ, Ding Y (2009). Clinical analysis of radio-chemotherapy for patients with locally advanced nasopharyngeal carcinoma. Chin J Cancer Prev and Treatment.

[4] Yin H, He ZG (2010). Clinical observation of 78 nasopharyngeal carcinoma patients treated with intensity modulated radiotherapy. J Med Res.

[5] Lin CG, Deng XW, Huang J, Wen ZX, Xiao DY, Lin LF (2004). A study of accuracy and reproducibility in conformal radiotherapy for nasopharyngeal carcinoma. J Oncol.

[6] Wang F, Yang SS (1999). Research on setup repeatability using electronic portal image device. Chin J Radi Oncol.

[7] Fan NB, Xia TY, Sun QX, Zhong F, Yuan D (2000). Comparison of effects of different mask labelling methods on precision of repeated positioning. Chin J Radiation Oncol.

[8] Huang SF, Zhu XZ, Xu JH, Jiang XS, Zhang YQ (20l0). Progress of intensity modulated radiation therapy in treatment of nasopharynx cancer. Chongqing Med J.

[9] Su T, Chen H (2011). Analysis of intensity-modulated radiotherapy on 116 cases of nasopharyngeal carcinoma. Chin Clin Oncol.

[10] Wang ZW, Wang MZ, Yang B, Chu JJ (2011). Changes of volume dose of normal organs in IMRT for nasopharyngeal carcinoma. J Clin Medi in Pra.

[11] Stroom JC, Boer HC, Huizenga HK (1999). Inclusion of geometrical un-certainties in radiotherapy treatment planning by means of coverageprobability. Radiat Oncol Biol Phys.

[12] Bai LL, Tian HR, Yao T, Liu QF, Zhang ZC, Wang Q (2010). Set-up errors in three-dimensional conformal radiation therapy of thoracic neoplasm. J Mod Oncol.

[13] He XH, Huang JL, Chen LX, Huang SM, Liang JB, Chen GZ (2004). Precise positioning technique of fractionated stereotatic radiotherapy for patients with head and neck cancer. Bulletin of Chin Cancer.

[14] Luo XL, Wang XC, Ren P (2012). Analysis of the disposition error on head-neck-shoulder mask in intensify treatment of nasopharynx cancer. Mod Hospital.

[15] Luo W, Lu TX (2002). Three-dimensional conformal radiation therapy for nasopharyngeal carcinoma. China Cancer.

[16] Zhang P, Wu SX, Xie CY, Jin XC (2005). Comparison study of IMRT treatment techniques for nasopharyngeal carcinoma. Chin J Medi Phy.

[17] Wang YR (2014). Clinical effect of intensity modulated radiotherapy treatment in treating patients with advanced nasopharynx cancer. Medi J Chin Peoples' Health.

[18] Ahmad S, Vlachaki M, Teslow TN, Amosson CM, McGary JE, Teh BS (2015). Impact of setup uncertainty in the dosimetry of prostate and surrounding tissues in prostate cancer patient treated with peacock/IMRT. Med Dosim.

